# The Effectiveness of Laser-Activated Irrigation on the Apical Microleakage Qualities of MTA Repair HP and NeoMTA Plus in Simulated Immature Teeth: A Comparative Study

**DOI:** 10.3390/ma13153287

**Published:** 2020-07-23

**Authors:** Serenad Çırakoğlu, Buket Baddal, Aylin İslam

**Affiliations:** 1 Department of Pediatric Dentistry, Faculty of Dentistry Near East University, 99138 Nicosia, North Cyprus; serenad.genc@gmail.com; 2 Department of Medical Microbiology and Clinical Microbiology, Faculty of Medicine, Near East University, 99138 Nicosia, North Cyprus; buket.baddal@neu.edu.tr; 3 Desam Institute, Near East University, 99138 Nicosia, North Cyprus

**Keywords:** antimicrobial, microleakage, laser, MTA HP, immature teeth

## Abstract

There are limited data regarding the potential effect of erbium, chromium: yttrium–scandium–gallium–garnet (Er,Cr:YSGG) laser-activated irrigation (LAI) on the microleakage qualities of calcium silicate-based cements. The objective of the present study was to assess the effect of LAI on the microleakage qualities of MTA Repair HP (MTA-HP) and NeoMTA Plus (Neo) used in root-end filling and to compare the antimicrobial effectiveness of MTA- HP. Two experimental sets were conducted: antimicrobial activity (agar diffusion test/at 24, 48 h) and microleakage (glucose leakage model/at 1st, 10th, 20th days). Antimicrobial activities of MTA-HP, Neo, Biodentine, ProRoot and MTA Angelus were evaluated, and inhibition zones were observed not only against a range of Gram-positive and Gram-negative bacteria but also against yeast at 48h. For microleakage evaluation, fifty teeth were prepared to simulate the clinical situation where the root-tips (apex) are open, and randomly divided into two experimental groups (n = 20/group) according to the cement type (*MTA-HP* and *Neo*), and two control (n = 5/group) groups. Each experimental group was further divided into two subgroups (n = 10/group) with respect to LAI: *MTA-HP*, *L-HP*, *Neo*, *L-Neo*. A statistical difference was only detected between Neo and L-HP groups on day 1. Subsequently, MTA-HP exhibited superior microleakage quality compared to Neo in the short-term. Er,Cr:YSGG laser-activated irrigation could be used as a reliable technique without creating adverse effects on the sealing abilities of MTA Repair HP and NeoMTA Plus.

## 1. Introduction

Three or more years are required for the development and apical closure of permanent teeth after eruption [1]. Traumatic injuries and severe caries within this critical period commonly result in pulpal necrosis and periapical periodontitis [1]. In this situation, major drawbacks associated with traditional root canal treatments arise, consisting of fractures or inadequate apical seal due to thin dentinal walls and wide-open apices [2,3]. The apexification method is extensively used for an incomplete root in teeth with necrotic pulp. Thereby, calcified tissue was apically formed to provide more suitable conditions for traditional root canal filling and to remove the risk of the over-extension of root canal filling to the periapical tissues [4]. Calcium hydroxide (CH) has been the most widely used and accepted material in apexification for decades. Although CH has proven to be highly successful in complacent patients, long-term CH apexification generates a number of drawbacks over time, such as long and multiple appointments, the difficulty of coronal seal integrity in appointment intervals, poor sealing capacity and setting problems [5,6]. It is possible to address all of the aforementioned problems with bioactive endodontic cements (BEC_S_) such as mineral trioxide aggregate (MTA) [7]. Conventional MTA cements are calcium silicate-based materials consisting of Portland cement supplemented with bismuth oxide (radiopacifier) [8]. Recently, the popularity of a single-visit apexification technique using MTA has increased, as it presents advantageous features including biocompatibility, dimensional stability, natural remineralization ability, hard tissue formation (apical barrier) ability in necrotic immature teeth and bacteriostatic effect [9,10]. However, concerns associated with MTA such as tooth discoloration, long setting time and poor handling still exist. Therefore, in order to circumvent all these, new formulations and products of BEC_S_ have been developed by researchers and manufacturers without altering the bioactivity and biocompatibility of MTA [11]. In this context, NeoMTA Plus and MTA Repair HP with new radiopacifiers have been introduced. NeoMTA Plus is based on a tricalcium silicate material consisting of tantalum oxide (Ta_2_O_5_) as a radiopacifier to prevent discoloration [12]. MTA Repair HP has been presented as a new formulation of White MTA. MTA Repair HP is newly launched bioactive cement with high plasticity and handling, in which calcium tungstate (CaWO_4_) has been used as a radiopacifier instead of bismuth oxide to improve the physico-chemical and biomechanical properties of the material. The liquid state of this product is obtained by the addition of an organic plasticizer (to gain plasticity and thus increase its manipulation) into distilled water [13,14]. These bioactive materials are mainly recommended for the dental pulp treatments (pulp capping, pulpotomy), apexification, apexogenesis, root-end filling and repair of root canal resorption [15].

The apical microleakage and sealing qualities of calcium silicate-based cements are primary considerations for the treatment of necrotic pulps to provide an apical barrier that blocks bacterial invasion and diffusion of bacterial products from root canal pathway into the periapical tissues [16,17]. However, the physicochemical and apical seal properties of root-end filling materials could be adversely affected and this can be correlated with manifold reasons such as acidic local microenvironment induced by bacterial metabolic products, the permeability of apical root dentin, or unavoidable contact with different irrigation solutions used for smear layer removal and disinfection of the root canal system [18,19,20]. Therefore, these adverse effects, which especially arise in the course of root canal irrigation, may be more marked with the use of device-combined irrigation procedures.

Recently, laser-activated irrigation (LAI) with particularly erbium lasers (Er:YAG/2980 nm and Er,Cr:YSGG/2780 nm) have become more popular with their effective dentinal smear layer removal and root canal disinfection abilities [20,21]. The main mechanism of LAI is expounded with transient cavitation effect, which allows the formation of vapor bubbles through the absorption of laser energy by water. These strong cavitation bubbles in the liquid expand during the pulse and subsequently implode, creating shock waves. This process may result in deeper cleaning and disinfection along the root canal [22,23]. Even though the effects of LAI and endodontic irrigation solutions on the bond strengths of calcium silicate cements have been investigated in several studies [24,25], the potential effect of particularly LAI on the sealing abilities of such biomaterials has not been sufficiently clarified. Hence, the present study aimed to assess the effect of LAI on the apical microleakage qualities ofMTA Repair HP and NeoMTA Plus used in root-end filling, and to evaluate their antimicrobial effectiveness with various calcium silicate-based cements.

## 2. Materials and Methods

### 2.1. Ethical Approval

All subjects gave their informed consent for inclusion before they participated in the study. The study was conducted in accordance with the Declaration of Helsinki, and the protocol was approved by the Ethics Committee of Near East University (NEU/2019/71/864).

The study protocol was conducted in two main experimental sets: antimicrobial activity and microleakage.

### 2.2. Antimicrobial Agar Diffusion Test (ADT)

Agar diffusion test was conducted to evaluate the antimicrobial activity of MTA Repair HP (Angelus, Londrina,Brasil) and NeoMTA Plus (NuSmile, Huston, TX, USA) comparing with three calcium silicate-based cements: Biodentine (Septodont Saint-Maur-des-Fosses Cedex, France), ProRoot MTA (Dentsply, Tulsa Dental Specialities,Konstantz, Germany), and MTA Angelus (Angelus, Londrina,Brasil). The cements used for the ADT were freshly mixed according to the manufacturer’s recommendations and applied to the agar plates. Detailed chemical compositions of used cements are given in Table 1.

Antimicrobial activities of calcium silicate-based cements were evaluated against *Staphylococcus aureus* (ATCC 25923), *Enterococcus faecalis* (ATCC 29212), *Pseudomonas aeruginosa* (ATCC 27853), and *Candida albicans* (ATCC 90,028). All tested microorganisms were cultured on blood agar except for *C. albicans,*which was cultured on Sabouraud dextrose agar (Oxoid) at 37 °C for 24 h. Three to four single colonies were picked and re-suspended in a 5 mL sterile phosphate-buffered saline and the inoculum was adjusted to equalize the turbidity to the 0.5 McFarland Standard. Agar diffusion tests were performed on Mueller-Hinton agar plates. Six equidistant wells of 5 mm depth and 3 mm diameter were punched by using a sterile agar gel puncher. The microbial suspensions were inoculated onto the plates using a sterile cotton-tipped applicator to achieve a lawn of growth. After inoculation, freshly mixed sealers were placed into the punched wells. The plates were incubated at 37 °C with 5% CO_2_ for 24 h. Subsequently, the diameter of the inhibition zones around each material was measured by the same operator in two perpendicular locations by using a millimetric ruler with an accuracy of 0.5 mm. Plates were returned to the incubator for another 24 h and the diameter of the inhibition zones were measured again at 48 h. The size of the inhibition zone was calculated as follows: Size of inhibition zone = (diameter of halo−diameter of the specimen) × ½. The mean diameter of the inhibition zone was statistically analyzed to assess the antimicrobial activity of the tested cements. Each microorganism was tested in triplicates. All assays were repeated three times to ensure reproducibility. 

### 2.3. Preparation of Simulated Immature Teeth Models 

A total of fifty extracted human maxillary central and lateral incisors were selected according to the selection criteria for this study. Only the teeth without resorption, extensive carious lesions, root anomalies, calcifications, previous root-canal treatment, posts, crowns, crack or fracture line were included in this study. Tissue remnants and calculus were removed mechanically from selected teeth by a periodontal scaler. Following steam autoclave sterilization, the teeth were washed with distilled water and kept in normal saline solution (Vacoliter, Baxter, Tekirdağ, Turkey) at 4 °C.

Selected teeth were decoronated 3 mm above the pulpal floor with diamond discs (Diabor, İstanbul, Turkey) under water cooling. Following decoronation, a #15 K-file (VDW, Munich, Germany) was inserted into the root canals until the visual apical foramen to standardize root canal lengths. The working length of each root was adjusted as 10 mm by resecting the root ends. The root canals were shaped with ProTaper files until F5 size (x-smart Plus, DENTSPLY, Ballaigues, Switzerland) to simulate the open apex and inner root canal structure of the immature teeth. The apical tips of root canals were standardized using a unicore driller (1.2 mm diameter, Ultradent, , İstanbul, Turkey). The root canals were irrigated using 5 mL of 1.5% NaOCl and 5 mLof saline solution. As the final step, all specimens were irrigated with 5 mL of 17% ethylene diamine tetra acetic acid (EDTA, Endo-Solution, Cerkamed, Wola, Poland) for removal of smear layer and washed with distilled water. Subsequently, all specimens were dried with paper points.

### 2.4. Determination of Groups for Microleakage Evaluation and Laser-Activated Irrigation 

The prepared teeth were randomly divided into two main experimental groups according to the type of cement (1) MTA Repair HP (n = 20); (2) NeoMTA Plus (n = 20) and two control groups (positive and negative, n = 5/each group). The cements were freshly mixed according to the manufacturer’s recommendations and placed in a retrograde direction to the root canals with an MTA carrier (Angelus, Dovgan, Canada). The thick end of moistened paper point was inserted into the canals to condense the cement as a 3 mm-thick apical plug. Following the radiographical correction of the proper application of the apical plugs, all specimens were wrapped in wet gauze and stored at 37 °C, 100% humidity for 1 week.

Thereafter, the experimental groups were further divided into two subgroups (n = 10/each group) with respect to laser- activated irrigation (LAI).

In subgroup one of NeoMTA Plus (Neo), each root canal was irrigated with 5 mL of 1.5% NaOCl using 30-gauge needle irrigation (NaviTip^TM^ Tips, Ultradent Products, Turkey). In subgroup one of MTA Repair HP (MTA-HP), each root canal was irrigated with the same procedure as in the Neo group.

In subgroup two of NeoMTA Plus (L-Neo), 5 mL of 1.5% NaOCl was activated by the Er,Cr:YSGG laser (Water-lase MD, Biolase, Irvine, CA, USA) at a wavelength of 2780 nm with 200 µm diameter, length of 21 mm, 0.55 calibration factor of radial firing tip (Endolase, Biolase Technology). The laser parameters were adjusted as 1.5 W of power, noncontact mode, a repetition rate of 20 Hz. Each specimen was treated with four repeated irradiations of 15 s, with 5 s intervals. RFT 2 tip was positioned 5 mm apically from the orifice. In subgroup two of MTA Repair HP (L-HP), each root canal was subjected to the same LAI procedure as the L-Neo group. Total irrigation time was adjusted as 2 min in all experimental groups.

Negative and positive control groups were designed to test the efficacy of the experimental system. In the negative control group, the MTA Angelus plug was applied to each root canal. In the positive control group, the prepared root canals were left unfilled. No irrigation was performed for both control groups. 

### 2.5. Glucose Leakage Model 

The glucose leakage model was based on Xu et al.’s endodontic leakage model [26]. At first, all surfaces of the teeth, except for root canal orifice and apical foramen in positive control and experimental groups, were coated with nail varnish, different to the negative control group in which the roots were completely coated. Then, the teeth were agglutinated to the end of an Eppendorf tube with cyanoacrylate where 2 mm of the apices was exposed outside. A plastic tube was conducted to the root canal orifice. Subsequently, the samples including the tooth, Eppendorf, and plastic tube were transferred in a sterile glass bottle. The side edges of the plastic tube were fixed to the Eppendorf tube with the aid of cyanoacrylate glue. Later, two holes were made on the glass bottle: the first one was for the plastic tube to pass through and the second hole was for providing atmospheric pressure inside the glass. A total of 5 mL of 1 mol/L glucose solution was injected into the Eppendorf tube through the plastic tube until the top of the solution was 14 cm higher than the top of canal orifice to create a hydrostatic pressure (1.5 kPa). A total of 5 mL of 0.2% NaN_3_ was added to the lower part of the model (glass bottle) to prevent decomposition of leaked glucose by the proliferation of microorganisms. Root apices of teeth were immersed in this NaN_3_ solution. After seal and control of all junctions, the model was transferred to an incubator with 100% humidity at 37 °C. The amount of glucose leakage was measured on the 1st, 10th, and 20th days.

### 2.6. Measurement of Microleakage

A 15 µmL aliquot of the solution was drawn from the glass bottle using a micropipette on the 1st, 10th, and 20th days. After drawing the sample, 15 µmL of fresh 0.2% NaN3 was added to the glass bottle reservoir to maintain a constant volume. The samples were analyzed with a Glucose Colorimetric Assay Kit (Cayman Chemicals, MI, USA) according to the instructions of the provider. Absorbance was measured at 520 nm wavelength using a microplate spectrophotometer (Versa Max, Molecular Device, Sunnyvale, ABD). Two blinded independent evaluators conducted the spectrophotometric determination of glucose concentration. The results of leakage in all groups were calculated as mmol/L at each timepoint.

### 2.7. Statistical Analysis

Power analysis of sample size was calculated as 50 patients by using G*Power (Ver.3.1.9.4) with 80% power at an alpha level of 0.05. Comparable data groups were analyzed by two-way analysis of variance (ANOVA) and Tukey’s post-hoc test was applied to obtain pair-wise comparisons. Graphpad Prism software (version 8.1.1) was used.

## 3. Results

### 3.1. Antimicrobial Activity

**Figure 1:**
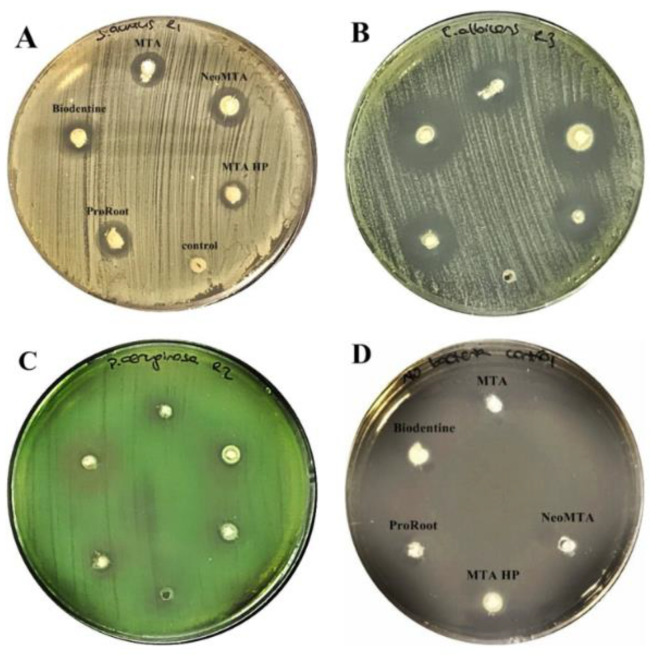
Zone of inhibition of five calcium silicate-based cements at 24 h against (**A**) *S*. *aureus*, (**B**) *C*. *albicans*, (**C**) *P*. *aeruginosa*, (**D**) No bacteria/control.

**Figure 2:**
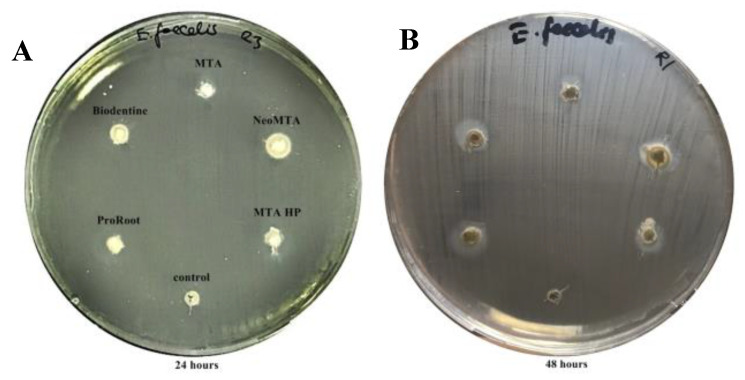
Zone of inhibition of five calcium silicate-based cements against *E. faecalis*. (**A**) inhibition zones of all calcium silicate-based cements at 24 h, (**B**) inhibition zones of all calcium silicate-based cements at 48 h.

All tested calcium silicate-based cements had inhibitory effects against *C. albicans*, *S.aureus,* and *P.aeruginosa* at 24 h as demonstrated by the inhibition zones. The zones of microbial growth inhibition for all tested cements against *C. albicans*, *S.aureus, P. aeruginosa* at 24 h are presented in Figure 1A–C.the uninoculated control plate showed no growth, ensuring that the tested materials were not contaminated, and this is presented in Figure 1D. No inhibitory activity was exhibited by any of the tested materials against *E. faecalis* within 24 h. At the end of the 48-h incubation period, a slight inhibition was detected by all of the five tested materials against *E. faecalis*. A comparison of inhibitory zones against *E. faecalis* at 24 h and 48 h is given in Figure 2. No statistically significant alterations in inhibition zones were observed between 24 h and 48 h against other microorganisms [Table S1: Mean and standard deviation values of inhibition zones at 24 h/48 h].

When inhibition zones at 24 h were compared across all calcium silicate-based cements per microorganism, NeoMTA Plus (11.58 ± 0.3) exhibited significantly better antimicrobial activity against *S. aureus* compared to MTA Angelus (9.5 ± 0.5), MTA Repair HP (8.33 ± 0.6) and Biodentine (8.5 ± 1.3) (*p* < 0.05, *p* < 0.001, *p* < 0.001, respectively) but no statistically significant differences were observed between MTA Angelus and ProRoot MTA (10.83 ± 0.5) activity. There were no statistically significant differences between the inhibitory effects exerted by any of the calcium silicate-based cements against other tested microorganisms.

**Figure 3:**
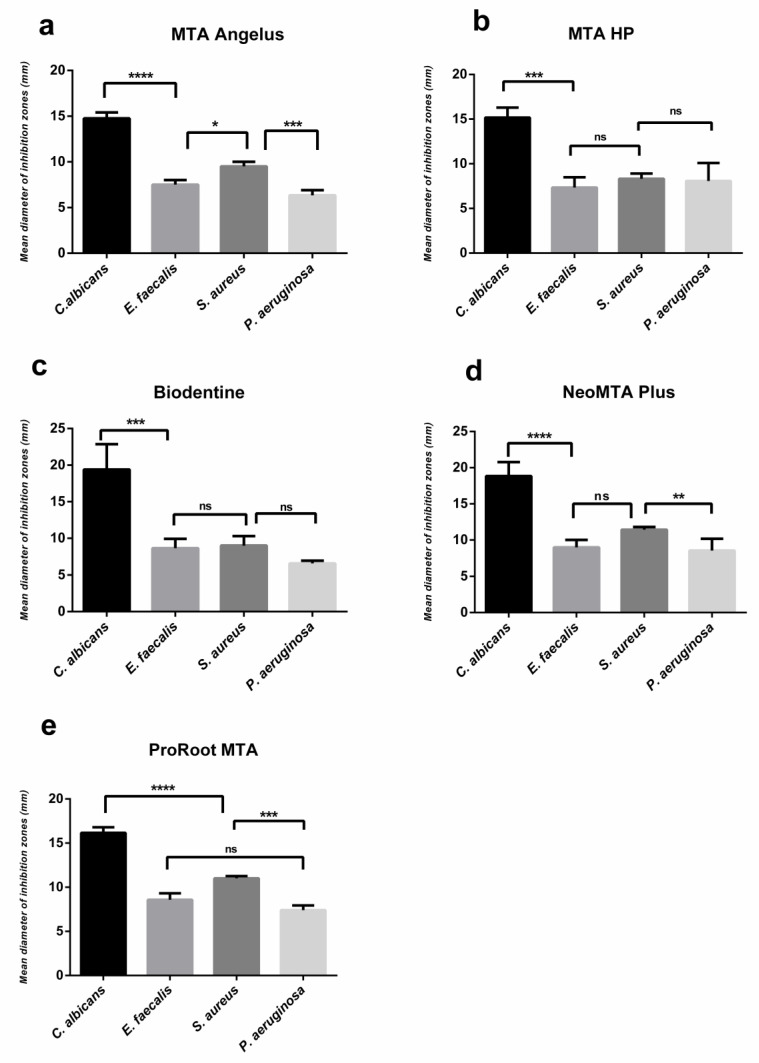
Inhibitory effect of each tested cement against per microorganism at the end of 48h. Results are expressed as mean values + SD (**** *p* < 0.0001; ***, *p* < 0.001; **, *p* < 0.01; *, *p* < 0.05; ns: no significance). (**a**) Inhibitory effect of MTA Angelus, (**b**) Inhibitory effect of MTA HP, (**c**) Inhibitory effect of Biodentine, (**d**) Inhibitory effect of NeoMTA Plus, (**e**) Inhibitory effect of ProRoot MTA.

When the mean diameters of inhibitions zones at 48 h were compared across all microorganisms per calcium silicate-based cements, all tested cements exhibited the highest inhibitory effect against *C. albicans.* MTA Angelus had significantly higher activity against *S. aureus* compared to *E. faecalis* and *P. aeruginosa* (*p* < 0.05, *p* < 0.001, respectively); whereas, in MTA Repair HP and Biodentine groups, no statistical differences were observed among three microorganisms (*p* > 0.05), except for *C. albicans*. The inhibitory effect of NeoMTA Plus was significantly higher against *S. aureus* than *P. aeruginosa* (*p* < 0.01), and ProRoot MTA demonstrated a similar activity which was higher against *S. aureus* than *P. aeruginosa* (*p* < 0.001). The antimicrobial activities of each tested cement against all microorganisms at 48h are shown in Figure 3.

### 3.2. Evaluation of Microleakage

**Figure 4:**
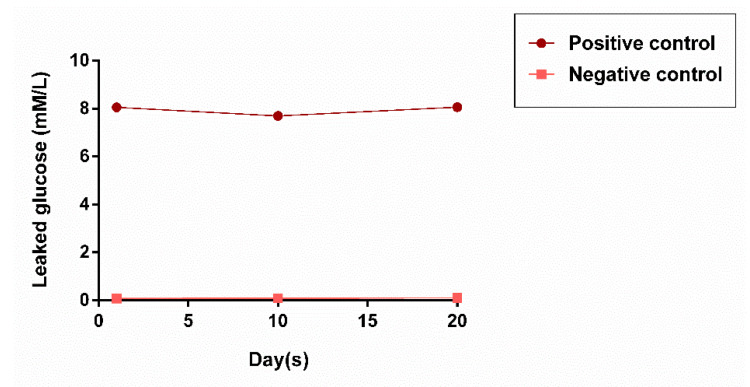
Mean glucose concentrations in control groups throughout the experimental period.

The mean values of leaked glucose in all experimental groups are presented in Table 2. Obtained data were initially checked for normality. Since all data were not normally distributed, Kruskal–Wallis and Dunn’s post-hoc tests were used for the analysis of differences between the groups. The highest values of leaked glucose were detected in the positive control group in all periods. No leakage of glucose was observed in the negative control group throughout the experiment (Figure 4).

**Figure 5:**
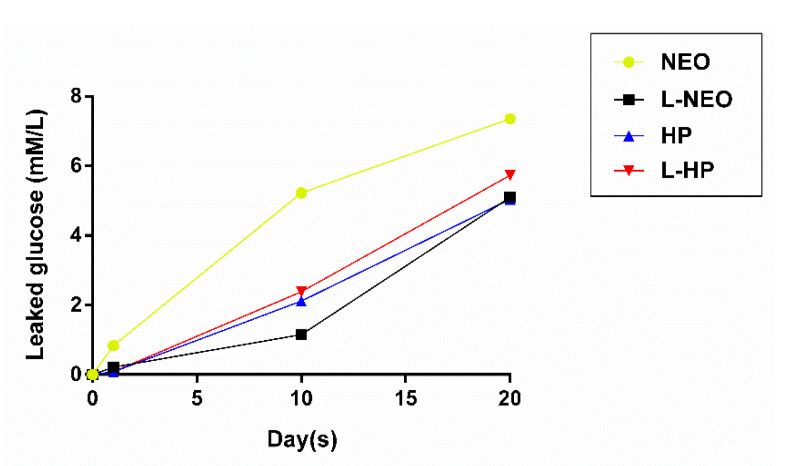
Mean glucose concentrations in all groups throughout the experimental period. Neo, NeoMTA Plus; L-Neo, NeoMTA Plus with laser-activated irrigation; HP, MTA Repair HP; L-HP, MTA Repair HP with laser -activated irrigation**.**

**Figure 6:**
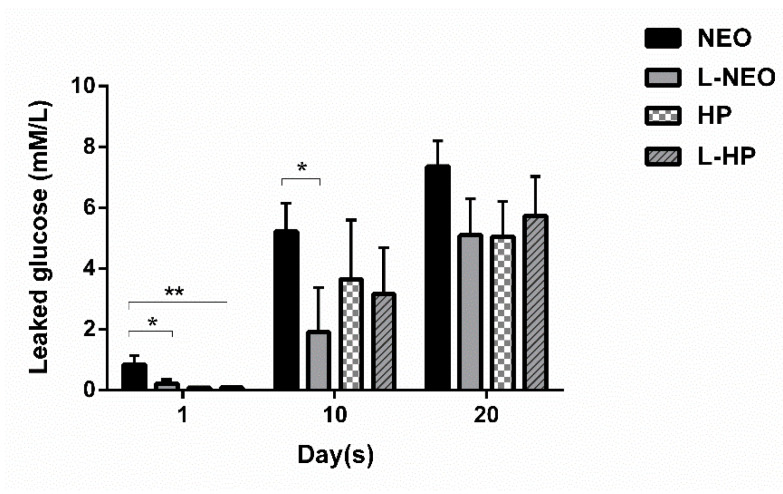
Glucose leakage across experimental groups at 1st, 10th, and 20th days. Results are expressed as mean values + SEM. Kruskal–Wallis, and Dunn’s post-hoc test was used for statistical analysis to compare the results across groups at 1st, 10th, and 20th days. (**, *p* < 0.01; *, *p* < 0.05; no asterisks, not significantly different).

An increase in levels of glucose leakage was observed in all experimental groups and was directly proportional with time (Figure 5). According to intergroup analyses among experimental groups, there were significant differences in microleakage at different timepoints. The L-Neo group exhibited significantly less microleakage than the Neo group on day 1 (*p* < 0.05) and day 10 (*p* < 0.05). While all groups demonstrated less leakage compared to the Neo group at alltimepoints, a statistically significant difference was only detected between Neo and L-HP groups on day 1(*p* < 0.01). Intergroup analyses of each experimental group at alltimepoints are shown in Figure 6.

According to the intragroup analysis of the Neo group, the lowest amount of glucose leakage (0.835 ± 0.973) was observed at the end of day 1. The amount of leakage was statistically significant compared to day 10 (5.220 ± 2.939, *p* < 0.01) and day 20 (7.356 ± 2.660, *p* < 0.001). No significant differences were detected between day 10 and day 20 of the Neo group (*p* > 0.05). In the L-Neo group, the greatest amount of glucose leakage was on day 20 (5.099 ± 3.752). The amount of leakage on day 20 was significantly higher when compared to that of day 10 (1.908 ± 4.665, *p* < 0.05) and day 1 (0.219 ± 0.398, *p* < 0.01).

**Figure 7:**
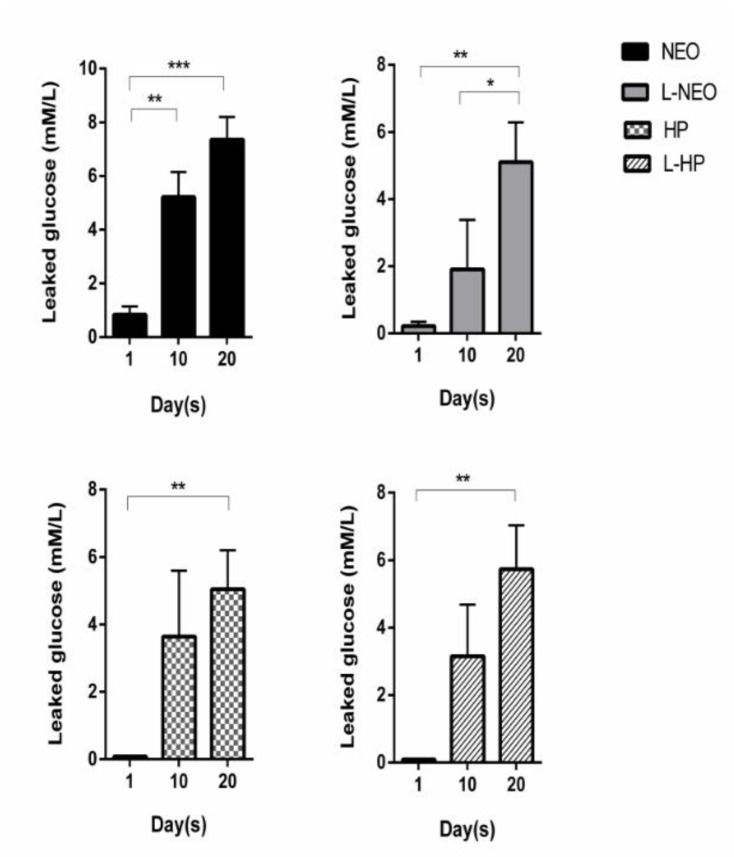
Glucose leakage per experimental group for 20 days. Results are expressed as mean values + SEM. Two-way ANOVA followed by Tukey’ s post-hoc test was used for statistical analysis to compare the results per group at 1st, 10th, and 20th days (***, *p* < 0.001; **, *p* < 0.01; *, *p* < 0.05; no asterisks, not significantly different).

Similar leakage patterns and mean values were observed in the MTA-HP and L-HP groups. Mean values of glucose leakage in both MTA-HP and L-HP groups on day 20 (5.043 ± 3.663; 5.729 ± 4.114, respectively) were significantly higher (*p* < 0.01) compared to the mean values (0.083 ± 0.005; 0.087 ± 0.019, respectively) on day 1 of the experiment. Intragroup analyses of each experimental group through experiment timepoints are shown in Figure 7.

## 4. Discussion

The apical microsealing ability of bioactive endodontic cements (BEC_S_) in the one-visit apexification treatment of necrotic immature permanent teeth is a fundamental issue for the future clinical success of treatment. Recently, the endodontic leakage characteristics of BEC_S_ applied as root-end filling material have come into prominence worldwide. First, the antimicrobial properties of MTA Repair HP and NeoMTA Plus were tested and compared with other three different calcium silicate-based cements (Biodentine, ProRoot MTA, andMTA Angelus) by using the agar diffusion test against a range of Gram-positive and Gram-negative bacteria as well as yeast, which are among the commonly isolated endo-perio pathogens. Secondarily, the efficacy of LAI on apical microleakage qualities ofMTA Repair HP and NeoMTA Plus, used as root-end filling materials on simulated immature teeth by a glucose leakage model, was assessed in the current study.

There are limited data regarding the microleakage qualities of MTA Repair HP as a root-end filling material in an apexification model in the literature. In this study, the apical microleakage activity of MTA Repair HP was compared with only NeoMTA Plus for use in root-end filling applications. The reasons for selecting NeoMTA Plus for comparison were its similar biocompatibility with MTA Repair HP on human dental pulp stem cells, high performance in bonding to root dentin by comparison with ProRoot MTA and Biodentine, andadequate clinical performance in terms of regeneration of cementum and periodontal ligament [14,27,28]. The sealing abilities of two tested materials could be linked to various influencing factors, such as the thickness of apical plug, immersion fluid, and the technique used for evaluation of apical microleakage. The thickness of the apical plug was based on Bani et al., and Çiçek et al., studies and was determined as 3 mm for each specimen in the current study. The thickness of apical plugs ranging between 3 to 6 mm exhibited an adequate sealing ability regardless of the tested materials in both studies [29,30]. Enzymatic glucose oxidase technique has become a preferred method for the evaluation of the sealing capacity of different materials [31] and the glucose leakage model used in this study isconsistent with the model described by XU et al., [26]. Features of this model, such as being cost-effective, allowing quantitative/sensitive measurements, providing clinical convenience and presenting non-invasiveness, are among the advantages which make it a preferred choice among investigators. Additionally, in contrast with the dye penetration test, it allows the monitoring of the seal to be traced for a long period without the need to destroy the specimen [32]. A gradual increase in glucose leakage was observed in all groups over 20 days during the experiment. MTA Repair HP demonstrated a lower microleakage in comparison to NeoMTA Plus in short-term evaluation. The differences observed between two tested BEC_S_ could be attributed to the physicochemical formulation of MTA Repair HP. The addition of a polymer plasticizer to the liquid structure of MTA Repair HP provides a better putty and homogeneous compositions that increase fluidity during manipulation procedures.Moreover, the low initial setting time of MTA Repair HP may lead to variations by increasing the effective biological responses of hydroxyapatite surfaces and creating hydrated gel forms to fill spaces between calcium silicate particles [33,34]. Hence, the short-term findings of the present study between MTA Repair HP and NeoMTA Plus are supported. 

In previous studies on disinfection efficacy, the smear layer removal and antibacterial effects of erbium lasers (Er,Cr:YSGG and Er:YAG) have been clarified by hydrokinetic and cavitation effects, which are based on the explosion of dentinal tubules by mineralized tissue ablation and also the possible formation of secondary bubbles due to previous laser pulses at the complex apical third part of root canals [35,36,37]. Distinct from the disinfection and smear layer removal effects of these lasers, the effect of LAI on the apical microleakage qualities of calcium silicate cements after retrograde filling has been investigated in the current study. According to the results of the current study, microleakage values in the LAI group of NeoMTA Plus (L-Neo) was lower than those of NeoMTA Plus (Neo) until day 10, even though the microleakage increased with time in both groups. Nevertheless, no differences were observed between MTA-HP and L-HP groups. In this respect, it should be interpreted that LAI did not show any adverse effects on the sealing capabilities of tested calcium silicate-based cements during the experimental period. On the contrary, the decrease in the microleakage values of NeoMTA Plus after LAI may be explained by the lower exposure (2 min) and concentrations (1.5%) of endodontic irrigation solutions. The short exposure time and low concentration of sodium hypochlorite in the present study (1.5%) was determined according to the study of Nagas et al., [25] and clinical considerations of American Association Endodontics (AAE) for regenerative procedures, respectively [38]. In a study by Betancourt et al., it was shown that the Er,Cr:YSGG LAI increased the bactericidal efficacy of 0.5% NaOCl against *E. faecalis* and also caused it to exhibit the same degree as in 5% NaOCl [39]. Eneide et al. also showed that antibiofilm efficacy of different irrigation solutions could be increased by various irrigation methods such as passive ultrasonic irrigation (PUI) and passive sonic irrigation (PSI), compared to theconventional needle irrigation technique [40]. Although a recent literature review reveals that new endodontic cement MTA Repair HP has successful physicochemical and biocompatible properties, such as including a plasticizer to simplify manipulation and insertion to the root canals [13,14,15], studies related to the root-end filling/apical sealing performance of MTA Repair HP, as well as to the effects of LAI on both tested calcium silicate cements,are very scarce. Therefore, the possibility of a comparison of the apical microleakage quality of these materials regarding LAI after retrograde filling with similar studies is limited.

Besides the sealing abilities of ideal calcium silicate-based cement, their antimicrobial activities are of paramount importance for the clinical success of treatment. Accordingly, in addition to the microleakage analysis, an antimicrobial agar diffusion test (ADT) was also performed in this study. ADT is considered to be the standard assay for the initial screening of antimicrobial activity. It is considered to be the most convenient method due to its simplicity and credibility for testing the antimicrobial properties of freshly mixed materials. MTA Repair HP and NeoMTA Plus were compared with other three calcium silicate-based cements (Biodentine, ProRoot MTA, MTA Angelus) to better evaluate its antifungal and antibacterial activities against *C. albicans, S. aureus, E. faecalis* and *P. aeruginosa*. In accordance with the study of Huang et al., [41], all five tested materials exhibited strong antifungal activity against *C. albicans*. All materials showed effective antibacterial activity against *S. aureus* and *P. aeruginosa* at 24 h. Although there was no clear indication of inhibitory activity of the materials tested against *E. faecalis* at 24 h, slight antibacterial activity was detected after 48 h. The ineffective antibacterial activity of all five tested materials against *E. faecalis* during 24 h may be associated with the resistant phenotype of *E. faecalis*. Despite the high alkaline pH levels of the tested calcium silicate-based cements, their alkalinity was inadequate to alter the microenvironment for inhibition of growth of this bacterium for the 24-hperiod [42]. The inhibitory activity against *E. faecalis* at the end of 48h may be due to an increase in the pH level of the environment around the tested cement materials, which resulted in the appearance of inhibition zones. This phenomenon was described by Torabinejadet al., [43] who reported that the pH of MTA is 10.5 at the time of mixing, but 12.9 after 3 h. In a study by McHugh et al. [44], the authors also showed that *E. faecalis* is unable to survive at pH values of 11.5 or higher. The ineffective inhibitory activity against *E. faecalis* at 24 h may also be due to the insensitivity of the preferred antibacterial test in the current study. Even though ADT is considered as the most convenient method, it may not be adequately sensitive and rapid to detect so-called viable but non-culturable (VBNC) bacteria in agar culture media [45] compared to direct contact test (DCT). The lack of a comparison of the antimicrobial effects of cements by ADT with the DCT method is a limitation of this study.

## 5. Conclusions

Among the tested parameters of the present study, MTA Repair HP exhibited a reduced microleakage quality compared to NeoMTAPlus, particularly in the short-term, the endodontic management of an immature permanent tooth using MTA Repair HP and NeoMTA Plus as an apical barrier exhibited similar acceptable antimicrobial and apical microleakage activity as the long term. Er,Cr:YSGG laser-activated irrigation could be used as a reliable technique without creating adverse effects on the sealing abilities of MTA Repair HP and NeoMTA Plus. The development of apexification models that simulate clinical situations better is required for further detailed investigations with larger sample sizes.

## Figures and Tables

**Table 1:** Chemical compositions of tested materials.

Material	Chemical Compositions	Manufacturer
Biodentine	Powder: Tricalcium silicate (3CaO.SiO_2_), dicalcium silicate (2CaO.SiO_2_), calcium carbonate (CaCO_3_), calcium oxide (CaO), and zirconium oxide (ZrO_2_). Liquid: Water, calcium chloride (CaCI_2_), Hydrosoluble polymer	Septodont Saint-Maur-des-Fosses, CEDEX, France
ProRoot MTA	Powder: Tricalciumsilicate (Ca_3_SiO_5_), dicalcium silicate (CaSiO_4_), tricalciumaluminate (3CaO.Al_2_O_3_), tetracalciumaluminoferrite (4CaO.Al_2_O_3_Fe_2_O_3_), free calcium oxide (CaO), bismuth oxide (Bi_2_O_3_). Liquid: Water (H_2_O)	Dentsply, Tulsa Dental Specialities, Germany
NeoMTA Plus	Powder: Tricalcium silicate (Ca_3_SiO_5_), Dicalcium silicate (Ca_2_SiO_4_) and Tantalum oxide (Ta_2_O_5_). Liquid: Water (H_2_O) and proprietary polymers	NuSmile, Houston TX USA
MTA Repair HP	Powder: Tricalciumsilicate (Ca_3_SiO_5_), dicalcium silicate (Ca_2_SiO_4_), tricalciumaluminate (3CaO.Al_2_O_3_), calcium oxide (CaO), and calcium tungstate (CaWC_4_). Liquid: Water and polymer plasticizer	Angelus, Londrina, Brasil
MTA Angelus	Tricalcium silicate, dicalcium silicate, tricalcium aluminate, tetracalciumaluminoferrite, bismuth oxide (MSDS)	Angelus, Londrina, Brasil

**Table 2:** Mean and standard deviation values of leaked glucose in four experimental groups (mM/L).

Time (Days)	NeoMTA Plus	L-NeoMTA Plus	MTA Repair HP	L-MTA Repair HP
1	0.835 ± 0.973	0.219 ± 0.398	0.083 ± 0.005	0.087 ± 0.019
10	5.220 ± 2.939	1.908 ± 4.665	3.644 ± 6.164	3.153 ± 4.836
20	7.356 ± 2.660	5.099 ± 3.752	5.043 ± 3.663	5.729 ± 4.114

## Supplementary Materials

The following are available online at https://www.mdpi.com/1996-1944/13/15/3287/s1, Table S1: Mean and standard deviation values of inhibitions zones at 24 h/48 h.
