# Supplementary material for: The Effectiveness of Laser-Activated Irrigation on the Apical Microleakage Qualities of MTA Repair HP and NeoMTA Plus in Simulated Immature Teeth: A Comparative Study
Source: Materials (Basel). 2020 Jul 23;13(15):3287. doi: 10.3390/ma13153287 (PMC7436115; doi:10.3390/ma13153287)
Supplement: Supplementary file 1 [file materials-13-03287-s001.pdf]

materials

Supplementary Materials

The Effectiveness of Laser-Activated Irrigation on The Apical Microleakage Qualities of MTA Repair HP and NeoMTA Plus in Simulated Immature Teeth: A Comparative Study

Serenad Çırakoğlu [1], Buket Baddal [2,3], Aylin İslam[1, 3,*]

[1] Department of Pediatric Dentistry, Faculty ofDentistryNear East University, 99138, Nicosia, North Cyprus; serenad.genc@gmail.com

[2] Department of Medical Microbiology and Clinical Microbiology, Faculty of Medicine, Near East University, 99138, Nicosia, North Cyprus; buket.baddal@neu.edu.tr

[3] Desam Institute, Near East University, 99138, Nicosia, North Cyprus

* Correspondence: aylin.islam@neu.edu.tr; Tel: +90-(392)-680-20-30; Fax: +90-(392)-680-20-25

Received: 18 June 2020; Accepted: 17 July 2020; Published: 23 July 2020

Table S1. Mean and standard deviation values of inhibition zones at 24 h/48 h.

**Type of Calcium Silicate Cements**	**Species of Microorganisms**							
	C.albicans		*P.aeruginosa*		*E.faecalis*		*S. aureus*	
	24 h	48 h	24 h	48 h	24 h	48 h	24 h	48 h
Biodentine	19.25 ± 3.36	19.42 ± 3.44	6.167 ± 0.28	6.58 ± 0.38	0	8.66 ± 1.25	8.5 ± 1.32	9 ± 1.29
ProRoot MTA	15.75 ± 0.43	16.17 ± 0.62	7.083 ± 1.23	7.41 ± 0.52	0	8.58 ± 0.72	10.83 ± 0.52	11 ± 0.25
NeoMTA Plus	18.5 ± 1.8	18.33 ± 1.94	7.167± 0.52	8.58 ± 1.58	0	9 ± 1	11.58 ± 0.28	11.42 ± 0.38
MTA Repair HP	15.5 ± 1.32	15.17 ± 1.12	6.5 ± 1.09	8.08 ± 2.03	0	7.33 ± 1.15	8.333 ± 0.57	8.33 ± 0.57
MTA Angelus	14.75 ± 0.66	14.42 ± 0.72	6.333 ± 0.57	6.91 ± 0.14	0	7.5 ± 0.5	9.5 ± 0.5	9.83 ± 0.76
Negative control	0	0	0	0	0	0	0	0

© 2020 by the authors. Licensee MDPI, Basel, Switzerland. This article is an open access article distributed under the terms and conditions of the Creative Commons Attribution (CC BY) license (http://creativecommons.org/licenses/by/4.0/).
